# Comparative Efficacy of Endotracheal Tube Holders Versus Traditional Securing Methods in the Intensive Care Unit: A Systematic Review and Meta‐Analysis

**DOI:** 10.1111/nicc.70196

**Published:** 2025-10-23

**Authors:** Shu‐Fen Lu, Wen‐Ju Yang, Shu‐He Huang, Shin‐Shang Chou, Shang‐Sin Shiu, Man‐Jyun Chen, Ching‐Ching Sheng, Ping‐Han Hsieh

**Affiliations:** ^1^ Department of Nursing Taipei Veterans General Hospital Taipei Taiwan; ^2^ School of Nursing, National Yang Ming Chiao Tung University Taipei Taiwan; ^3^ Department of Nursing, College of Nursing National Yang Ming Chiao Tung University Taipei Taiwan; ^4^ Division of Gastroenterology and Hepatology, Department of Medicine Koo Foundation Sun Yat‐Sen Cancer Center Taipei Taiwan; ^5^ School of Medicine, College of Medicine National Yang Ming Chiao Tung University Taipei Taiwan

**Keywords:** AnchorFast, endotracheal tube holder, endotracheal tube securing method, medical device‐related pressure injury, meta‐analysis

## Abstract

**Background:**

An endotracheal tube (ETT) can be securely positioned using a holder or traditional securing method. The holder is a one‐piece moulded design, and traditional securing methods usually involve manual fixation using adhesive tape or bandages. ETT holders are superior to traditional securing methods in reducing medical device‐related pressure injuries (MDRPIs) and the incidence of tube dislodgement. However, evidence from previous meta‐analyses is highly heterogeneous.

**Aim:**

To compare the efficacy of ETT holders and traditional securing methods through a systematic review and meta‐analysis of MDRPIs and dislodgement rates.

**Study Design:**

Four major databases were searched for relevant randomised controlled trials (RCTs) and non‐RCTs. Two authors independently extracted data and assessed the quality of the studies. A meta‐analysis was conducted using a random‐effects model to estimate the risk ratios and risk differences with 95% confidence intervals. The primary and secondary outcomes were the total number of MDRPIs and tube dislodgements, respectively. Subgroup analyses were performed based on the severity of the condition and nutritional status.

**Results:**

A systematic search yielded 3737 references, among which seven met the inclusion criteria. These included three RCTs, two quasi‐experimental cohorts and two observational cohorts (*n* = 3025). Pooled analyses showed a significant reduction in total MDRPI risk and a decreasing trend of composite tube dislodgements for ETT holders compared with traditional securing methods. Subgroup analysis indicated that ETT holders were less effective than traditional securing methods in preventing MDRPIs in patients who were not malnourished or with less severe conditions.

**Conclusion:**

Compared with traditional securing methods, ETT holders significantly reduced MDRPI incidence and showed a decreasing trend in dislodgement. However, these effects were not evident in patients with less severe conditions or in those who were not malnourished.

**Relevance to Clinical Practice:**

This study highlights that ETT holders significantly reduced MDRPIs, particularly in patients with more severe disease (APACHE II score ≥ 20). Unplanned dislodgements were consistently less frequent with ETT holders; however, the preventive effect on MDRPIs was less evident in patients with less severe disease or better nutritional status. Therefore, disease severity and nutritional status should guide the choice of ETT securement methods and support ICU nurses in enhancing airway safety, minimising complications and standardising care.

**Impact Statements**

- What is known about the topic
  ○ Medical device‐related pressure injuries (MDRPIs) and tube dislodgements are common in critically ill patients.
  ○ Endotracheal tube (ETT) holders reduce composite events, such as pressure injuries and tube dislodgement, compared with traditional securing methods.
  ○ Previous meta‐analyses indicated that ETT holders decrease pressure injuries compared with traditional securing methods, despite significant heterogeneity among studies.
- What this paper adds
  ○ This study demonstrated that the use of ETT holders reduces MDRPIs and potentially decreases composite events of tube dislodgement compared with traditional securing methods. However, ETT holders may not be superior to traditional securing methods in preventing MDRPIs in patients with less severe conditions or who are not malnourished.

## 1 Introduction

Insertion of an endotracheal tube (ETT) is an essential intervention for critically ill patients in the intensive care unit (ICU) to maintain airway and respiratory functions during mechanical ventilation. Securing the ETT is crucial for achieving effective oral hygiene, preventing tube dislodgement and maintaining airway patency in patients. However, the methods used are key factors in ETT‐related skin injuries [1], such as those affecting the lips, oral cavity and face. These injuries often conform to the shape of the device and are classified in the guidelines as medical device‐related pressure injuries (MDRPIs) [2]. Traditionally, ETTs are manually secured by nursing staff using adhesive tape or bandages, a practice referred to as traditional securing methods. Subsequently, a commercial ETT holder featuring a one‐piece moulded design that includes a gliding tube, lip stabiliser and tube wrap was developed to reduce ETT complications.

Previous randomised controlled trials (RCTs) have shown that commercial devices result in fewer composite events of lip ulcers and facial tears, as well as lower rates of dislodgement than adhesive tapes [3]. A meta‐analysis showed that the incidence of MDRPIs was lower with commercial devices (AnchorFast) than with adhesive tape [4]. However, some issues in previous meta‐analyses require further clarification.

The need for this updated meta‐analysis stems from the limitations of a previous study [4]. An earlier meta‐analysis reviewed only four studies and identified several methodological issues that required correction. These include inconsistent definitions of MDRPIs and errors in the coding numbers that were identified during the review. Moreover, the high heterogeneity reported (Higgins statistic [*I* ^2^], 93.2%) was solely attributed to selection bias in one study [5], without further exploration through subgroup or sensitivity analysis. Additionally, an earlier meta‐analysis did not adequately consider potential confounders, such as patients' nutritional status or the severity of conditions related to immobility, which are critical factors influencing MDRPIs. These gaps highlight the need for a more comprehensive and accurate evaluation of the current evidence.

Therefore, this study aimed to synthesise the latest evidence on methods of securing ETTs, specifically comparing ETT holders with traditional securing methods, while addressing previous issues, to provide more definitive evidence for enhancing nursing care for critically ill patients in the ICU.

## 2 Methods

This study was conducted according to the Preferred Reporting Items for Systematic Reviews and Meta‐analyses Guidelines (see Appendix A) [6]. The protocol for this review was registered with PROSPERO (CRD42024553541) after the searches had commenced; however, registration was completed before the searches were conducted, acknowledging its retrospective nature.

### 2.1 Eligibility Criteria

This systematic review and meta‐analysis was designed to identify randomised controlled trials (RCTs) and non‐RCTs comparing the effects of two different methods of securing ETTs in patients admitted to the ICU, specifically focusing on the use of ETT holders or the traditional securing methods most commonly used in regular daily care. The eligible trials provided information adhering to the following criteria: (a) patients admitted to the ICUs with ETTs and (b) use of two different methods of securing the ETT (holder or traditional securing methods, including adhesive tapes or bandages). References were excluded if they met at least one of the following criteria: (a) comparison of only different ETT holders or only different traditional securing methods instead of comparing both types; (b) studies on patients not admitted to the ICU; (c) studies using simulation manikins or cadavers; (d) studies with patients in the prone position; (e) studies of nasal ETTs or tracheostomies; or (f) lack of clearly stated clinical outcomes.

### 2.2 Source of Evidence and Selection of Evidence

Potential references were identified through keyword searches of the Cochrane CENTRAL (trial registry), EMBASE, PubMed and CINAHL Plus databases, with searches conducted across the full text of articles. Relevant keywords were applied to free text and medical subject headings (e.g., MeSH terms for PubMed and EMTREE terms for EMBASE) using terms such as synonyms for ICU, holders and traditional securing methods. The Boolean operators ‘OR’ and ‘AND’ were used to connect search returns of synonyms and identify intersections between two sets of keywords, specifically, synonyms of ICU and methods of securing ETTs. No filters were applied based on publication time, language or study design restrictions. The most recent search was conducted in May 2024, and the detailed search strategy is presented in Table S1. The research team meticulously and manually searched the reference lists of relevant articles in associated journals to identify additional studies that investigated the effects of ETTs and their measurement methods on treatment outcomes in patients admitted to the ICU. The identified references were imported into EndNote X20 (Clarivate Analytics) for eligibility screening. After the automatic exclusion of duplicates using the ‘Find Duplications’ function in EndNote, the remaining references were screened by two researchers (S.F.L. and P.H.H.) based on the predefined eligibility criteria. In cases of disagreement regarding inclusion, the researchers convened to discuss the reasons and reached a consensus through dialogue.

### 2.3 Data Extraction and Quality Evaluation

Two researchers (S.F.L. and P.H.H.) independently extracted the data into Excel (Microsoft, USA) files from each target paper and cross‐verified the details of the included trials, focusing on information related to the inclusion and exclusion criteria, randomisation and non‐randomisation processes, methods of securing ETTs, sex, age, severity of condition, nutritional status and the effects of the methods of securing ETTs. The primary outcomes were ETT‐related MDRPIs, including all pressure injuries at sites possibly related to ETTs and methods of securing them. Secondary outcomes included MDRPIs at different anatomical sites (lips, oral cavity and face) and ETT dislodgement. Since the effects of the methods of securing ETTs, including MDRPIs or ETT dislodgements, were the outcomes of interest in this review, the events and sample sizes in each group were extracted with a notation of zero for studies reporting no events during the treatment and follow‐up periods. Data were extracted using the intention‐to‐treat (ITT) rather than the per‐protocol (PP) approach to mitigate bias, adhere to the spirit of intention‐to‐treat rather than treatment adherence, and ensure greater applicability to real‐life scenarios. If only PP data were available, the outcomes were presented in the main text. In the event of a disagreement during data extraction, the two researchers jointly conducted a triple check. Based on the extracted information regarding the study design and outcome data, two researchers independently appraised the risk of bias using the Cochrane risk of bias tool (RoB2) for RCTs and ROBINS‐I for non‐RCTs.

### 2.4 Data Synthesis and Analysis

Descriptive data were tabulated to provide an overview of patient demographics (age and sex), aetiology and baseline characteristics possibly associated with pressure injuries, such as the severity of the condition, nutritional status, smoking status and use of steroids or vasopressors. Quantitative data were synthesised using Review Manager 5.4. The outcomes were dichotomous for both MDRPIs and tube dislodgements; therefore, the risk ratio (RR) or risk difference (RD) was used as the effect measurement for events with zero notations in the results. Data were pooled using the Mantel–Haenszel method. Given the inevitable clinical heterogeneity, quantitative data were synthesised using a random‐effects model. Statistical heterogeneity among studies was assessed using the Cochran Q‐test (*p* < 0.10) and *I* ^2^ ≥ 50% indicating a substantial level of heterogeneity with a 95% confidence interval (CI). Statistical significance was set at *p* < 0.05. To address concerns regarding clinical heterogeneity and enhance the clarity of the results, meta‐regression and sensitivity analyses were conducted using R software through RStudio. Subgroup analyses were performed based on the severity of the condition, nutritional status, type of ETT holder versus traditional securing method, study design, repositioning frequency of the ETT and severity of MDRPIs. Publication bias was assessed using funnel plots if the meta‐analysis included more than 10 studies.

### 2.5 Certainty Evaluation of the Synthesis

The Grading of Recommendations, Assessment, Development, and Evaluation (GRADE) working group guidelines were applied using GRADEpro [7] to construct an evidence profile table for systematic reviews, focusing on important outcomes differentiated by study design, including RCTs and non‐RCTs. The team members further evaluated the pooled findings of important outcomes according to the risk of bias, inconsistency, indirectness, imprecision, publication bias, large effect size, dose–response gradient and effect of plausible residual confounding. The results were confirmed and agreed upon during the research team meetings.

## 3 Results

**FIGURE 1:**
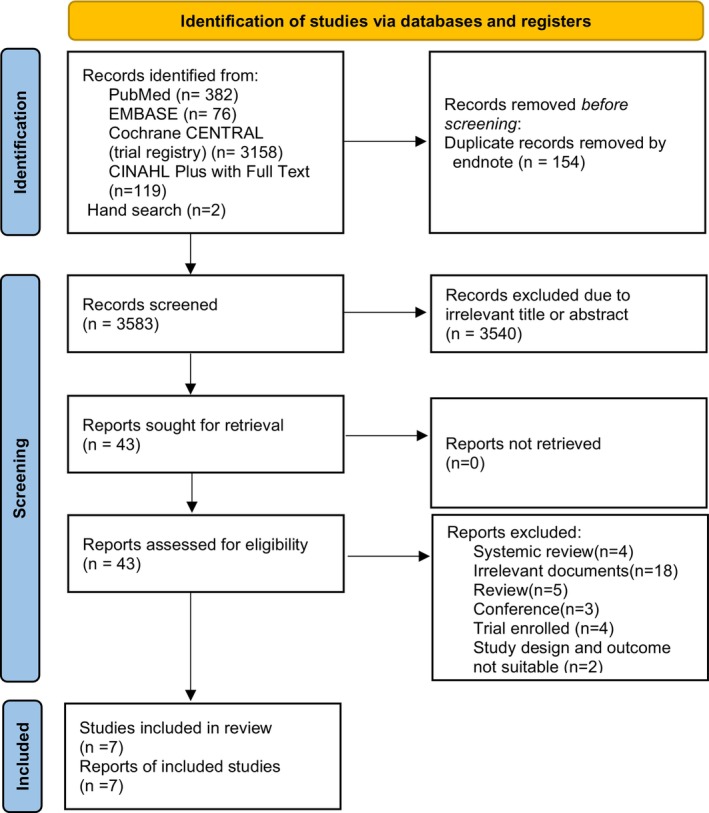
Flowchart of trial selection investigating the treatment effect of endotracheal tube (ETT) holders versus traditional securing methods.

A comprehensive search strategy combining systematic and manual methodologies initially identified 3737 references for review. Following automated deduplication processes (*n* = 154), screening for irrelevant titles or abstracts (*n* = 3540) and a thorough review of the full texts (*n* = 43), seven records were found to be eligible based on the predefined criteria. These seven references corresponded to reports from three RCTs [3, 8, 9], two quasi‐experimental studies [10, 11] and two observational cohort studies [5, 12]. All identified studies were subsequently incorporated into the synthesis (Figure 1).

### 3.1 Characteristics

A collective cohort of 3025 individuals diagnosed with a critical illness who required ETT insertion within 1 day and remained in situ for more than 24 h without facial or oral injuries was admitted to the ICU. Data for the analysis were drawn from seven studies, which served as the basis for the synthesis. The patients were from the United States, Australia, the Netherlands, Israel and Turkey, with data spanning 1994 to 2020.

The treatment modalities administered in the studies included ETT holders (1,135 patients) and traditional securing methods (1,890 patients). Five studies used AnchorFast as the holder, whereas the other two used different commercial devices, one of which was not specified. Regarding traditional securing methods, five studies used the tape method and two used the bandage method. The baseline characteristics revealed an equal distribution of sexes in most studies, with an age range of approximately 50–70 years. Three studies reported patients with a body mass index ranging from 24 to 41, indicating that they were overweight or obese. In addition, three studies revealed that most patients had a severe condition, with an Acute Physiology and Chronic Health Evaluation II (APACHE II) score of approximately ≥ 20 [3, 8, 9] and two studies reported a potential malnutrition group and related MDRPIs [5, 9]. The proportions of smokers, steroid users, vasopressor users, individuals with peripheral vascular disease and those with type 2 diabetes mellitus varied among the studies, with limited information and no related MDRPIs provided (Table 1 and Table S4).

**TABLE 1:** Baseline characteristics of patients.

Author year	Country	Design	Sample size H/TSM	Age^#^	Sex male *n* (%)	Comparison H/TSM	Location of MDRPI	APACHE II^#^	Severe disease (H/TSM)	Nutrition status
Kaplow et al. [10]	USA	QED	81/30	53.8 ± 18.3	55 (50%)	Comfit Holder/Lillihei harness	Lip, face, back of neck	NA	NA	NA
Embregts et al. [12]	Netherlands	Prospective cohort	80/69	NA	NA	Anchor Fast/Lillehel harness	Lip	NA	NA	NA
Hampson et al. [5]	Australia	Retrospective cohort	596/1412	(h) 58.6 (49.3–73.6) TSM: 53.2 (39.2–67.1)	H: 11 (52.4) TSM: 17 (81%)	Anchor Fast/ Fixsond (Bandage)	Lip and oral	NA	NA	Malnutrition H/TSM: 2 (9.5%)/6 (28.6%)
Landsperger et al. [3]	USA	RCT	250/250	(h) 53.2 ± 16.4 TSM: 58.5 ± 16.1	H: 77 (50.3%) TSM: 79 (54.5%)	Anchor Fast/Cloth tapes	Lip and face	H:26.0 ± 8.9 TSM: 27.4 ± 8.7	250/250	NA
Coyer et al. [8]	Australian	RCT	21/21	55.8 (48.0–68.0)*	27 (60%)	Anchor Fast/Adhesive tapes	No information	Median: 23 (18–27)*	21/21	NA
Kuniavsky et al. [11]	Israel	QED	77/78	(h) 71.04 ± 15.73 TSM: 70.76 ± 15.6	(h) 44 (57%) TSM: 42 (53.8%)	Anchor Fast/Cotton tape	Lip, face, ear	NA	NA	NA
Genc et al. [9]	Turkey	RCT	30/30	51.03 ± 10.29	34 (57%)	Holder/Bandage	Oral	H/TSM 0‐10:1/0; 10‐20:8/6; 20‐35:21/22; ≥35:0/2	21/24	Nutrition risk scoring H/TSM: High risk: 23/26; Low risk: 7/4

Abbreviations: H, holder; NA, not available; QED, quasi‐experimental design; TSM, traditional securing methods.

* Only includes patients in the endotracheal tube group.

^#^ Median (IQR), Mean ± SD.

The within‐study definitions of MDRPIs, primarily located on the lip, face and oral areas, are shown in Table 1. Five studies focused on lip ulcers [3, 5, 10, 11, 12], three on facial ulcers [3, 10, 11] and two on oral ulcers [5, 9]. Only three studies provided data on tube dislodgement [3, 10, 12]. Composite dislodgements were defined as ETT repositioning by > 1 cm or self‐extubation to compare the stability of ETT holders with that of traditional securing methods. The risk of bias is described in Tables S2 and S3, with further details in Tables S4 and S5.

### 3.2 MDRPIs and Dislodgements

**FIGURE 2:**
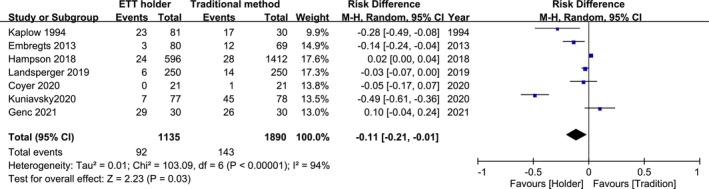
Forest plot of seven studies assessing total medical device‐related pressure injury (MDRPI) events (*N* = 3025).

Seven studies involving 3025 patients were analysed [3, 5, 8, 9, 10, 11, 12]. The pooled results demonstrated a significant reduction in the risk of total MDRPIs (RD, −0.11; 95% CI, −0.21 to −0.01; *p* = 0.03) with ETT holders compared to traditional securing methods, but with high heterogeneity (*I* ^2^ = 94%) (Figure 2).

**FIGURE 3:**
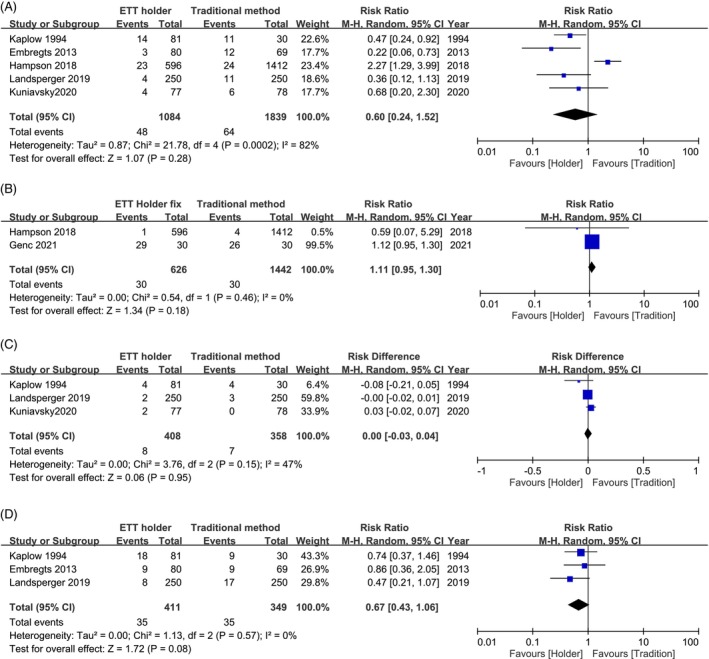
Forest plot for secondary outcomes: Medical device‐related pressure injuries (MDRPIs) in (A) lip ulcers, (B) oral ulcers, (C) facial ulcers and (D) endotracheal tube dislodgements.

MDRPIs are classified as lip, oral or facial ulcers. Five of the seven studies (*n* = 2923) focused on lip ulcers. The pooled results demonstrated a reduced trend in the risk of lip ulcers (RR, 0.6; 95% CI, 0.24–1.52; *p* = 0.28) but with high heterogeneity (*I* ^2^ = 82%) when comparing ETT holders with traditional securing methods. Additionally, no significant difference was observed in oral ulcers (RR, 1.1; 95% CI, 0.95–1.3; *p* = 0.18) in two of the seven studies (*n* = 2068) and facial ulcers (RD, 0.00; 95% CI, −0.03 to 0.04; *p* = 0.95) in three of the seven studies (*n* = 766). In three of the seven studies (*n* = 760), the focus was on ETT dislodgement. The pooled results demonstrated a superior reducing trend in the risk of ETT dislodgement (RR, 0.67; 95% CI, 0.43 to 1.06; *p* = 0.08) with low heterogeneity (*I* ^2^, 0%) (Figure 3A,D).

### 3.3 Subgroup Analysis

**FIGURE 4:**
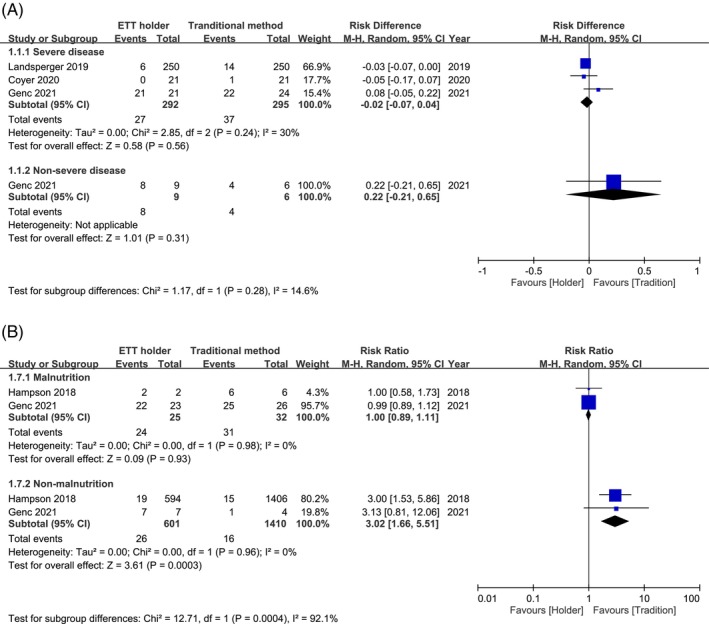
Subgroup analysis of total medical device‐related pressure injury (MDRPI) events stratified by (A) disease severity and (B) nutritional status.

Owing to the high heterogeneity observed in the total MDRPIs, a meta‐regression based on the publication year was conducted, which revealed no significant correlation with the total MDRPIs (*p* = 0.46) (Figure S2A). Further sensitivity and influence analyses based on the total MDRPI effects revealed persistently high heterogeneity (*I* ^2^ = 81%–94%) without evidence of a dominant bias from any single study (Figure S2B,C). Therefore, subgroup analyses were performed to examine potential confounding factors, including disease severity, nutritional status, frequency of ETT repositioning, study design and MDRPI grade. An APACHE II score of approximately 20 was used as the cutoff to distinguish between severe and non‐severe conditions. Malnutrition was defined using the nutritional risk score in one study [9], whereas direct data were provided in another study [5]. These subgroup analyses were conducted to explore the potential causes of the observed heterogeneity (Figure 4A,B). Three of the seven studies had information on severe disease status [3, 8, 9] (*n* = 587), and the pooled results demonstrated a reducing trend in the risk of total MDRPIs (RD, −0.02; 95% CI, −0.07 to 0.04; *p* = 0.56, *I* ^2^ = 30%) when comparing ETT holders with traditional securing methods. Two of the seven studies had information on malnutrition status (*n* = 2068) [5, 9]. The pooled results demonstrated no significant reduction in the risk of total MDRPIs in the malnutrition group (RR, 1.00; 95% CI, 0.89–1.11; *p* = 0.93, *I* ^2^, 0%) and a significant increase in the risk of total MDRPIs in the non‐malnutrition group (RR, 3.02; 95% CI, 1.66–5.51; *p* < 0.01, *I* ^*2*^, 0%) when comparing ETT holders with traditional securing methods. Three of the seven studies provided information on the frequency of repositioning the ETT (*n* = 2110), and the pooled results demonstrated no significant reduction in the risk of total MDRPIs in the holder group with a higher repositioning frequency (RD, 0.01; 95% CI, −0.02 to 0.05; *p* < 0.01, *I* ^2^, 17%). Subgroups of different holders, traditional securing methods, study designs and MDRPI grades showed persistently high heterogeneity (Figure S1A–D).

### 3.4 Certainty of Evidence

Three RCTs assessed using the RoB2 tool revealed that two studies had a high risk of bias owing to missing outcome data. In contrast, one study raised concerns regarding the randomisation process. The non‐RCTs evaluated using ROBINS‐I showed three studies with a serious risk and one with a moderate risk, mainly because of uncontrolled confounding factors (Tables S2 and S3). To assess publication bias, a funnel plot with Egger's test was performed for reference, even though the study did not include more than 10 studies; the results were not significant (Figure S3).

The GRADE evidence table (Figure S4) shows that the certainty of the evidence ranged from very low for RCT designs in two important outcomes to very low and low for non‐RCT designs, respectively. Certainty was downgraded mainly because of the risk of bias, inconsistency and imprecision. Regarding MDRPIs in RCTs, non‐RCTs and tube dislodgements in RCTs, the certainty was further downgraded because of imprecision from relatively small sample sizes or inconsistencies.

## 4 Discussion

When comparing ETT holders to traditional securing methods in patients admitted to the ICU, fewer total MDRPIs and a decreasing trend in ETT dislodgement were observed for ETT holders. Additionally, ETT holders may not be suitable for patients with less severe conditions or those who are less malnourished. This underscores the practical implications of using ETT holders versus traditional securing methods and offers guidance on selection based on patient severity and nutritional status to optimise nursing care and improve patient outcomes.

### 4.1 MDRPI

According to the American Association of Critical‐Care Nurses (AACN) guidelines, routine checks of oral and lip conditions every 2 h and repositioning of the ETT at regular intervals after ICU admission are recommended for the early detection and reduction of MDRPIs [13]. Securing ETT holders and traditional securing methods are important factors associated with MDRPIs. Previous studies on traditional securing methods have compared different fixation methods, including fixing bandages and adhesive tape [14], as well as manual techniques such as adhesives, twill or a simple bow for ETT fixation [15]. However, owing to the variety of taping and bandage methods for ETTs, which are experience‐ and dexterity‐dependent, there was heterogeneity in the fixation conditions related to the nurses' duties. This non‐standardised approach has led to a lack of unified recommendations based on AACN guidance. An ETT holder was designed for easy and standardised manipulation of oral ETTs. Several commercially available holders are available, with AnchorFast being the most frequently used [3, 5, 8, 11, 12]. Most holders share similar design features: a gliding tube for easy repositioning of the ETT while securing the tube, a lip stabiliser to prevent contact with the upper lip and reduce the risk of lip ulcers and a tube wrap to lock the tube in position and prevent dislodgement. We found a decrease in total MDRPIs when ETT holders were used compared to traditional securing methods, with a decreasing trend in lip ulcers but not in oral or facial MDRPIs (Figures 2 and 3A–C). This may be due to the function of the lip stabiliser and gliding tube in facilitating easier tube repositioning. A previous study discussed the force associated with the use of ETT holders versus traditional securing methods, noting that ETT holders may exert more stress on the face but offer a more secure fit with easier movement of the ETT [16]. However, there is no evidence of a higher incidence of facial tears in previous RCTs [3], as found in this review, although the results were obtained from only three studies. This may be due to the use of skin barrier pads designed to resist moisture and provide gentle protection to the skin when in contact with the face. Further analysis suggested a trend towards improved benefits for patients with severe conditions when using ETT holders compared to traditional securing methods (Figure 4A). Patients with severe conditions, who are possibly less awake and more immobile due to sedation and vasopressor drugs, are more prone to developing MDRPIs. In addition, critically ill patients have poor perfusion status and reduced healing ability, making them more susceptible to MDRPIs and infections. ETT holders have a greater potential to decrease the risk of MDRPIs in patients with severe conditions than traditional securing methods, possibly because of their more structured design, which includes easy repositioning, oral care, lip stabilisation and a face pad. Conversely, in patients without severe conditions who were not malnourished, those using traditional securing methods appeared to have fewer MDRPIs than those using ETT holders. This may result from the patients' increased awareness of the ETT, allowing greater activity and mobility, which induces greater friction and shear forces between the device and the facial tissues [17]. Unlike traditional securing methods, such as adhesive tape, which may shift slightly with skin movement and distribute pressure more evenly, ETT holders exert concentrated forces at fixed contact points, particularly around the mouth and cheeks. This localised pressure, combined with frequent facial movements or patient repositioning, may increase the risk of tissue damage. Although ETT holders may exert more stress on the face, traditional securing methods, such as adhesive tape, allow for slight shifts in skin movement, distributing pressure more evenly [18]. These factors may explain the higher incidence of MDRPIs observed in more mobile and less critically ill patients using ETT. Caution is warranted when applying these findings to choose securing methods for patients without malnutrition or severe illness, as conclusions were drawn from a limited number of studies [5, 9]. The sample size of patients without malnutrition was large (*n* = 2011); however, most of the data were obtained from a single study, limiting the generalisability of the results. Nonetheless, this study suggests a potential link between malnutrition, disease severity and the risk of MDRPIs, with lower heterogeneity in these subgroups. Further studies are needed to confirm these associations and provide stronger evidence for this.

### 4.2 Rates of Dislodgement of the ETT

Traditional methods for securing ETT, such as using tape or bandages, may lead to tube slipping during repositioning or when patient mobility increases. In contrast, ETT holders employ a tube‐wrap design to secure the tube, incorporating a sliding mechanism that allows repositioning without pushing or pulling the tube. These features help explain the observed trend of reduced dislodgement in this study. This underscores the practical advantages of ETT holders in maintaining secure tube positioning during nursing care.

### 4.3 Strengths of the Review

This review has several strengths. First, it included 3025 patients and updated the most recent evidence to clarify the possible sources of the previously reported high heterogeneity. Second, this review provides clear guidance for clinical practice in ICU settings by highlighting the advantages and limitations of ETT holders and traditional securing methods. These findings suggest that ETT holders may be more suitable for critically ill patients with severe conditions, offering improved stability and a reduced risk of MDRPIs. Conversely, for patients with less severe conditions or those who are not malnourished, traditional securing methods may be preferable because they reduce the risk of pressure‐related injuries. Finally, these findings provide evidence‐based recommendations for tailoring nursing care, optimising patient outcomes and improving the quality of care in critical care settings. This study provides a foundation for future prospective studies to generate more definitive evidence on this topic.

### 4.4 Limitations of the Review

First, the data were pooled from RCTs and non‐RCTs, raising concerns regarding potential bias. However, given that the enrolled RCTs demonstrated very low certainty of evidence (Figure S4), non‐RCT studies were included to complement the RCTs and provide a more comprehensive body of evidence [17]. Nevertheless, over 50% of the included studies had a high risk of bias (Tables S2 and S3). In contrast, many studies have reported patient loss to follow‐up during the study period in RCTs and potential confounding factors in non‐RCTs. In this study, we used an ITT approach with subgroup analysis to minimise possible heterogeneity between studies. This approach facilitated the identification of relatively clear evidence and provided important insights into its clinical use and potential for future study designs. Second, this study did not sufficiently account for differences in nursing care protocols, such as oral care practices and the timing of changes in ETT securement methods, owing to the limited available data. Qualitative information summarising these variations is presented in Table S4. In the ETT holder group, repositioning was performed every 2 h, as opposed to every 6 h with traditional securing methods. Hampson et al. reported that ETT holders should be replaced every 3–5 days [5]. Despite these differences, the results showed a higher overall incidence of MDRPIs in the traditional securing method group. To investigate this further, a subgroup analysis was conducted based on the repositioning frequency. Only three studies reported such data; however, the analysis revealed no significant differences in total MDRPIs (Figure S1D) [5, 8, 9]. This suggests that the primary cause of MDRPIs may not be solely attributable to differences in nursing care protocols. Third, the differences in methods between ETT holders and traditional securing methods and the severity of MDRPIs may have introduced bias. This study conducted a subgroup analysis to account for variations in fixation based on differences in the methods of securing ETTs and the severity of MDRPIs, which showed that fixation was not the main confounding factor (Figure S1B). Finally, in clinical practice, variability in patients' facial shapes and sizes poses a challenge because commercially available ETT holders are not designed to accommodate individual anatomical differences. In this study, we did not examine the relationship between facial shape and MDRPIs due to the limited available information. This can increase the risk of MDRPIs and cause discomfort in some patients. Future research and product development should prioritise the creation of adaptable or adjustable ETT holders to address this issue and improve patient safety and comfort.

## 5 Relevance to Clinical Practice

For critical care nurses in the ICU, secure ETT fixation is essential to prevent pressure injuries and unplanned dislodgement. This review suggests that ETT holders may be more effective than traditional securing methods in reducing dislodgement rates. The benefits of preventing MDRPIs appear to be more pronounced in patients with more severe disease (APACHE II score ≥ 20). Conversely, traditional securing methods (such as adhesive tape or bandages) may be suitable for patients with mild illnesses or those without malnutrition. These findings support the need for a tailored ETT security approach to enhance patient safety and optimise nursing care in critical care settings.

## 6 Conclusions

This review synthesises the latest evidence comparing the effectiveness of ETT‐securing methods with holders versus traditional securing methods among patients admitted to the ICU. These findings indicate that ETT holders significantly reduce the incidence of MDRPIs; however, this may not apply to patients with less severe conditions or those who are not malnourished. Additionally, ETT holders can decrease the frequency of ETT dislodgements compared to traditional securing methods. These results highlight the suitability of ETT holders and traditional securing methods for enhancing the safety of critically ill patients and improving ICU outcomes.

## Ethics Statement

This study was conducted according to the Preferred Reporting Items for Systematic Reviews and Meta‐Analyses guidelines.

## Consent

The authors have nothing to report.

## Conflicts of Interest

The authors declare no conflicts of interest.

## Supporting information


**Table S1:** Search strategy.
**Table S2:** Risk of bias (Cochrane Risk of Bias tool 2, [RoB 2]).
**Table S3:** Risk of bias (ROBINS‐I).
**Table S4:** Details of the included studies.
**Table S5:** Detailed outcomes of the included studies.
**Figure S1:** Subgroup analysis of total medical device‐related pressure injury (MDRPI) events by: (A) Study type, (B) Type of holder vs. traditional securing methods, (C) Pressure injuries above grade 2 only and (D) Frequency of repositioning.
**Figure S2:** (A) Meta‐regression by publication year, (B) sensitivity analysis and (C) influence analysis of total medical device‐related pressure injuries (MDRPIs).
**Figure S3:** Funnel plot of included studies (*N* = 7).
**Figure S4:** GRADE evidence table for patients in intensive care units receiving endotracheal tube (ETT) holders versus traditional securing methods (non‐holders).

## Data Availability

The data that supports the findings of this study are available in the Supporting Information of this article.

## Appendix A PRISMA 2020 Checklist

**Table:**

Section and topic	Item #	Checklist item	Location where item is reported
**TITLE**			
Title	1	Identify the report as a systematic review.	P1
**ABSTRACT**			
Abstract	2	See the PRISMA 2020 for Abstracts checklist.	P1
**INTRODUCTION**			
Rationale	3	Describe the rationale for the review in the context of existing knowledge.	P2‐3
Objectives	4	Provide an explicit statement of the objective(s) or question(s) the review addresses.	P3
**METHODS**			
Eligibility criteria	5	Specify the inclusion and exclusion criteria for the review and how studies were grouped for the syntheses.	P3; 2.1
Information sources	6	Specify all databases, registers, websites, organisations, reference lists and other sources searched or consulted to identify studies. Specify the date when each source was last searched or consulted.	P4; 2.2
Search strategy	7	Present the full search strategies for all databases, registers and websites, including any filters and limits used.	P4;2.2; Table S1
Selection process	8	Specify the methods used to decide whether a study met the inclusion criteria of the review, including how many reviewers screened each record and each report retrieved, whether they worked independently, and if applicable, details of automation tools used in the process.	P4; 2.2
Data collection process	9	Specify the methods used to collect data from reports, including how many reviewers collected data from each report, whether they worked independently, any processes for obtaining or confirming data from study investigators, and if applicable, details of automation tools used in the process.	P4; 2.3
Data items	10a	List and define all outcomes for which data were sought. Specify whether all results that were compatible with each outcome domain in each study were sought (e.g., for all measures, time points, analyses), and if not, the methods used to decide which results to collect.	P4; 2.4
	10b	List and define all other variables for which data were sought (e.g., participant and intervention characteristics, funding sources). Describe any assumptions made about any missing or unclear information.	P4; 2.3; 2.4
Study risk of bias assessment	11	Specify the methods used to assess risk of bias in the included studies, including details of the tool(s) used, how many reviewers assessed each study and whether they worked independently, and if applicable, details of automation tools used in the process.	P3‐4; 2.3 Table S2.1
Effect measures	12	Specify for each outcome the effect measure(s) (e.g., risk ratio, mean difference) used in the synthesis or presentation of results.	P5; 2.4
Synthesis methods	13a	Describe the processes used to decide which studies were eligible for each synthesis (e.g., tabulating the study intervention characteristics and comparing against the planned groups for each synthesis (item #5)).	P5; 2.4
	13b	Describe any methods required to prepare the data for presentation or synthesis, such as handling of missing summary statistics, or data conversions.	P5; 2.4
	13c	Describe any methods used to tabulate or visually display results of individual studies and syntheses.	P5; 2.4
	13d	Describe any methods used to synthesise results and provide a rationale for the choice(s). If meta‐analysis was performed, describe the model(s), method(s) to identify the presence and extent of statistical heterogeneity, and software package(s) used.	P5; 2.4
	13e	Describe any methods used to explore possible causes of heterogeneity among study results (e.g., subgroup analysis, meta‐regression).	P5; 2.4
	13f	Describe any sensitivity analyses conducted to assess robustness of the synthesised results.	P5
Reporting bias assessment	14	Describe any methods used to assess risk of bias due to missing results in a synthesis (arising from reporting biases).	P5; 2.3
Certainty assessment	15	Describe any methods used to assess certainty (or confidence) in the body of evidence for an outcome.	P5; 2.5
**RESULTS**			
Study selection	16a	Describe the results of the search and selection process, from the number of records identified in the search to the number of studies included in the review, ideally using a flow diagram.	P6; 3.1; Figure 1
	16b	Cite studies that might appear to meet the inclusion criteria, but which were excluded, and explain why they were excluded.	P6; 3.1; Figure 1
Study characteristics	17	Cite each included study and present its characteristics.	P6‐7;3.1; Table 1
Risk of bias in studies	18	Present assessments of risk of bias for each included study.	P7‐9;3.1; Tables S2 and S3
Results of individual studies	19	For all outcomes, present, for each study: (a) summary statistics for each group (where appropriate) and (b) an effect estimate and its precision (e.g., confidence/credible interval), ideally using structured tables or plots.	P7;3.2., Figures 2 and 3
Results of syntheses	20a	For each synthesis, briefly summarise the characteristics and risk of bias among contributing studies.	P7‐8;3.2,3.3; Figures 2, 3, 4
	20b	Present results of all statistical syntheses conducted. If meta‐analysis was done, present for each the summary estimate and its precision (e.g., confidence/credible interval) and measures of statistical heterogeneity. If comparing groups, describe the direction of the effect.	P7‐8; 3.2, 3.3; Figures 2, 3, 4
	20c	Present results of all investigations of possible causes of heterogeneity among study results.	P8;3.3; Figure 4A,B
	20d	Present results of all sensitivity analyses conducted to assess the robustness of the synthesised results.	P7, Figure S2A,B
Reporting biases	21	Present assessments of risk of bias due to missing results (arising from reporting biases) for each synthesis assessed.	P8; 3.4; Figure S3
Certainty of evidence	22	Present assessments of certainty (or confidence) in the body of evidence for each outcome assessed.	P8; 3.4; Figure S4
**DISCUSSION**			
Discussion	23a	Provide a general interpretation of the results in the context of other evidence.	P9‐12; 4.1, 4.2, 4.3
	23b	Discuss any limitations of the evidence included in the review.	P11‐12; 4.4
	23c	Discuss any limitations of the review processes used.	P11‐12; 4.4
	23d	Discuss implications of the results for practice, policy, and future research.	P2,9,11;4.3
**OTHER INFORMATION**			
Registration and protocol	24a	Provide registration information for the review, including register name and registration number, or state that the review was not registered.	P3 (CRD42024553541)
	24b	Indicate where the review protocol can be accessed, or state that a protocol was not prepared.	P3
	24c	Describe and explain any amendments to information provided at registration or in the protocol.	NA
Support	25	Describe sources of financial or non‐financial support for the review, and the role of the funders or sponsors in the review.	Title page P2‐3
Competing interests	26	Declare any competing interests of review authors.	Title page P2‐3
Availability of data, code and other materials	27	Report which of the following are publicly available and where they can be found: template data collection forms; data extracted from included studies; data used for all analyses; analytic code; any other materials used in the review.	Supplementary files

## References

[1] F. A. M. Saad , H. Ahmed , N. Rezk , and N. A. Kandeel , “Endotracheal Tube Nursing Care: Current Evidence,” Mansoura Nursing Journal 9, no. 1 (2022): 177–187, 10.21608/mnj.2022.259016.

[2] L. E. Edsberg , J. M. Black , M. Goldberg , L. McNichol , L. Moore , and M. Sieggreen , “Revised National Pressure Ulcer Advisory Panel Pressure Injury Staging System: Revised Pressure Injury Staging System,” Journal of Wound, Ostomy, and Continence Nursing 43, no. 6 (2016): 585–597, 10.1097/WON.0000000000000281. PMC5098472 27749790

[3] J. S. Landsperger , J. M. Byram , B. D. Lloyd , and T. W. Rice , “The Effect of Adhesive Tape Versus Endotracheal Tube Fastener in Critically Ill Adults: The Endotracheal Tube Securement (ETTS) Randomised Controlled Trial,” Critical Care 23, no. 1 (2019): 161, 10.1186/s13054-019-2440-7. 31064406 PMC6505126

[4] C. H. Moser , A. Peeler , R. Long , et al., “Prevention of Endotracheal Tube‐Related Pressure Injury: A Systematic Review and Meta‐Analysis,” American Journal of Critical Care 31, no. 5 (2022): 416–424, 10.4037/ajcc2022644. 36045034

[5] J. Hampson , C. Green , J. Stewart , et al., “Impact of the Introduction of an Endotracheal Tube Attachment Device on the Incidence and Severity of Oral Pressure Injuries in the Intensive Care Unit: A Retrospective Observational Study,” BMC Nursing 17 (2018): 4, 10.1186/s12912-018-0274-2. 29449786 PMC5806388

[6] J. P. Radua , “PRISMA 2020—An Updated Checklist for Systematic Reviews and Meta‐Analyses,” Neuroscience and Biobehavioral Reviews 124 (2021): 324–325, 10.1016/j.neubiorev.2021.02.016. 33596413

[7] G. Guyatt , A. D. Oxman , E. A. Akl , et al., “GRADE Guidelines: 1. Introduction‐GRADE Evidence Profiles and Summary of Findings Tables,” Journal of Clinical Epidemiology 64, no. 4 (2011): 383–394, 10.1016/j.jclinepi.2010.04.026. 21195583

[8] F. Coyer , J. L. Cook , W. Brown , A. Vann , and A. Doubrovsky , “Securement to Prevent Device‐Related Pressure Injuries in the Intensive Care Unit: A Randomised Controlled Feasibility Study,” International Wound Journal 17, no. 6 (2020): 1566–1577, 10.1111/iwj.13432. 32596937 PMC7948617

[9] A. Genc and T. Yildiz , “Impact of Two Distinct Endotracheal Tube Fixations on Pressure Ulcer Formation in the Intensive Care Unit: A Randomised Controlled Trial,” International Wound Journal 19, no. 6 (2022): 1594–1603, 10.1111/iwj.13757. 35088531 PMC9493224

[10] R. Kaplow and M. Bookbinder , “Comparison of Four Endotracheal Tube Holders,” Heart & Lung 23, no. 1 (1994): 59–66. 8150646

[11] M. Kuniavsky , E. Vilenchik , and A. Lubanetz , “Under (Less) Pressure ‐ Facial Pressure Ulcer Development in Ventilated ICU Patients: A Prospective Comparative Study Comparing Two Types of Endotracheal Tube Fixations,” Intensive & Critical Care Nursing 58 (2020): 102804, 10.1016/j.iccn.2020.102804. 32029382

[12] N. Embregts , P. Van Berkom , F. Van Beers , et al., “An Endotracheal Tube Fixation Device Which Reduces Lip Pressure Ulcers in Critically Ill Patients. Conference Abstract,” Intensive Care Medicine 39 (2013): S404, 10.1007/s00134-013-3095-5.

[13] AACN , AACN Procedure Manual for Progressive and Critical Care‐e‐Book (Elsevier Health Sciences, 2023).

[14] J. Seyedhosseini , M. Ahmadi , A. Nejati , et al., “Two Different Endotracheal Tube Securing Techniques: Fixing Bandage Versus Adhesive Tape,” Advanced Journal of Emergency Medicine 1, no. 1 (2017): e3, 10.22114/AJEM.v1i1.6. 31172055 PMC6548090

[15] H. M. Mohammed and M. S. Hassan , “Endotracheal Tube Securements: Effectiveness of Three Techniques in Orally Intubated Patients,” Egyptian Journal of Chest Diseases and Tuberculosis 64, no. 1 (2015): 183–196, 10.1016/j.ejcdt.2014.09.006.

[16] D. F. Fisher , C. T. Chenelle , A. D. Marchese , J. P. Kratohvil , and R. M. Kacmarek , “Comparison of Commercial and Noncommercial Endotracheal Tube‐Securing Devices,” Respiratory Care 59, no. 9 (2014): 1315–1323, 10.4187/respcare.02951. 24368866

[17] A. Gefen , P. Alves , G. Ciprandi , et al., “Device‐Related Pressure Ulcers: SECURE Prevention,” Journal of Wound Care 29, no. Sup2a (2020): S1–S52, 10.12968/jowc.2020.29.Sup2a.S1. 32067552

[18] J. M. Black , J. E. Cuddigan , M. A. Walko , L. A. Didier , M. J. Lander , and M. R. Kelpe , “Medical Device‐Related Pressure Ulcers in Hospitalised Patients,” International Wound Journal 7, no. 5 (2010): 358–365, 10.1111/j.1742-481X.2010.00699.x. 20561094 PMC7951307

