# Supplementary material for: Comparative Efficacy of Endotracheal Tube Holders Versus Traditional Securing Methods in the Intensive Care Unit: A Systematic Review and Meta‐Analysis
Source: Nurs Crit Care. 2025 Oct 23;30(6):e70196. doi: 10.1111/nicc.70196 (PMC12570123; doi:10.1111/nicc.70196)
Supplement: Supplementary file 1 — Table S1: Search strategy. Table S2: Risk of bias (Cochrane Risk of Bias tool 2, [RoB 2]). Table S3: Risk of bias (ROBINS‐I). Table S4: Details of the included studies. Table S5: Detailed outcomes of the included studies. Figure S1: Subgroup analysis of total medical device‐related pressure injury (MDRPI) events by: (A) Study type, (B) Type of holder vs. traditional securing methods, (C) Pressure injuries above grade 2 only and (D) Frequency of repositioning. Figure S2: (A) Meta‐regression by publication year, (B) sensitivity analysis and (C) influence analysis of total medical device‐related pressure injuries (MDRPIs). Figure S3: Funnel plot of included studies (N = 7). Figure S4: GRADE evidence table for patients in intensive care units receiving endotracheal tube (ETT) holders versus traditional securing methods (non‐holders). [file NICC-30-0-s001.docx]

**Supplementary Materials – Content**

**Table S1. Search strategy**

**Table S2. Risk of bias (Cochrane Risk of Bias tool 2, [RoB 2])**

**Table S3. Risk of bias (ROBINS-I)**

**Table S4. Details of the included studies**

**Table S5. Detailed outcomes of the included studies**

**Figure S1. Subgroup analysis of total medical device-related pressure injury (MDRPI) events by: (A) Study type (B) Type of holder vs traditional securing methods (C) Pressure injuries above grade 2 only (D) Frequency of repositioning**

**Figure S2. (A) Meta-regression by publication year (B) Sensitivity analysis (C) Influence analysis of total medical device-related pressure injuries (MDRPIs)**

**Figure S3. Funnel plot of included studies (N = 7)**

**Figure S4. GRADE evidence table for patients in Intensive Care Units receiving endotracheal tube (ETT) holders versus traditional securing methods (non-holders)**

**Table S1. Search strategy**

| Endotracheal tube fastener or AnchorFastTM or Endotracheal tube holder or Endotracheal tube fixator  **OR**  Adhesive tape or Transpore tapeTM or Urgosyval tape or TransporeTM White tape or Multipore tape or DuraporeTM tape or velcro tape or waterproof cloth tape or twill tape or cotton tape or gauze  **AND**  Intensive care unit or ICU | **Pubmed: 382** |
| --- | --- |
|  | **Cochrane advanced search: 3158** |
|  | **CINAHL Plus with Full Text: 119** |
| ('intensive care unit'/exp OR 'gicu' OR 'gicus' OR 'icu`s' OR 'close attention unit' OR 'combined medical and surgical icu' OR 'combined surgical and medical icu' OR 'critical care unit' OR 'general icu' OR 'intensive care department' OR 'intensive care unit' OR 'intensive care units' OR 'intensive therapy unit' OR 'intensive treatment unit' OR 'medical-surgery icu' OR 'medical/surgical icu' OR 'medical/surgical icus' OR 'medico-surgical icu' OR 'mixed medical and surgical icu' OR 'mixed surgical and medical icu' OR 'respiratory care unit' OR 'respiratory care units' OR 'special care unit' OR 'surgery/medical icu' OR 'surgical-medical icus' OR 'surgical/medical icu' OR 'unit, intensive care') AND ('endotracheal tube fixation device'/exp OR 'endotracheal tube fixation device' OR 'tracheal tube fixation device' OR 'adhesive tape'/exp OR 'medfix' OR 'medfix ez' OR 'neo-prep' OR 'adhesive tape' OR 'adhesive tapes' OR 'basic adhesive tape' OR 'non-sterile skin surface device adhesive' OR 'orthopaedic adhesive tape' OR 'orthopedic adhesive tape' OR 'silicone adhesive tape' OR 'skin surface device adhesive' OR 'skin surface device adhesive, non-sterile' OR 'skin surface device adhesive, sterile' OR 'waterproof adhesive tape') | **Embase: 76** |

**Table S2. Risk of bias (Cochrane Risk of Bias tool 2, [RoB 2])**

| **Study** | **Domain 1** | **Domain 2** | **Domain 3** | **Domain 4** | **Domain 5** | **Overall** |
| --- | --- | --- | --- | --- | --- | --- |
| **Landsperger 2019** | **Low risk** | **High risk^+^** | **High risk^#^** | **Some concerns^%^** | **Low risk** | **High risk** |
| **Coyer 2020** | **Low risk** | **Some concerns*** | **High risk^#^** | **Some concerns^%^** | **Low risk** | **High risk** |
| **Genc 2022** | **Some concerns**^a^ | **Low risk** | **Low risk** | **Low risk** | **Low risk** | **Some concerns** |

*Domain 1: Bias arising from the randomisation process*

*Domain 2: Bias due to deviations from intended interventions*

*Domain 3: Bias due to missing outcome data*

*Domain 4: Bias in measurement of the outcome*

*Domain 5: Bias in selection of the reported result*

** Non-blinded study, without no information about its relation to the trial context*

*^#^ High proportion of loss follow-up/exclusions (>20%), with a 3.4 likelihood that missingness depend on true values (possibly yes)*

*^%^ Outcome assessment likely influenced by knowledge of intervention (possibly not)*

*+ Non-blinded study, with no information about relation to the trial context; analysis not appropriate to estimate the effect (score range 2.6–2.7), with substantial impact on results*

***a*** *No information available regarding allocation concealment*

**Table S3. Risk of bias (ROBINS-I)**

| **Study** | **Domain 1** | **Domain 2** | **Domain 3** | **Domain 4** | **Domain 5** | **Domain 6** | **Domain 7** | **Overall** |
| --- | --- | --- | --- | --- | --- | --- | --- | --- |
| **Kaplow 1994** | **Serious risk*** | **Moderate risk^&^** | **Low risk** | **Moderate risk^%^** | **Moderate risk^+^** | **Moderate risk^!^** | **Low risk** | **Serious risk** |
| **Embregts 2011** | **Serious risk*** | **Low risk** | **Low risk** | **Moderate risk^b^** | **No information** | **Moderate risk^!^** | **Low risk** | **Serious risk** |
| **Hampson 2018** | **Moderate risk^a^** | **Low risk** | **Moderate risk^@^** | **Moderate risk^b^** | **Low risk** | **Low risk** | **Low risk** | **Moderate risk** |
| **Kuniavsky 2020** | **Serious risk*** | **Low risk** | **Low risk** | **High risk^^^** | **Low risk** | **Moderate risk^!^** | **Low risk** | **Serious risk** |

*Domain 1: Bias due to confounding*

*Domain 2: Bias in selection of participants into the study*

*Domain 3: Bias in classification of interventions*

*Domain 4: Bias due to deviations from intended interventions*

*Domain 5: Bias due to missing data*

*Domain 6: Bias in measurement of outcomes*

*Domain 7: Bias in selection of the reported result*

* Nutritional status and drug use were not considered as initial confounding factors.

a Sedation drugs and status were not considered as initial confounding factors

b Sedation drugs were not considered co-interventions.

& The Compfit holder group was discontinued early because it slipped easily.

% Different portions of sedated patients may reflected different levels of sedation drug co-intervention.

+ The proportion of missing data among the holder groups differed slightly across intervention groups.

! Outcome assessors were not blinded

^ Co-intervention factors were not considered.

@ Study design was retrospective

**Table S4. Details of the included studies**

| **Study** | **Kaplow 1994** | **Embregts 2011** | **Hampson 2018** | **Landsperger 2019** | **Coyer 2020** | **Kuniavsky 2020** | **Genc 2021** |
| --- | --- | --- | --- | --- | --- | --- | --- |
| **Included/Exclusion criteria** | 1. At least 18 years of age 2. Orally intubated within the last 8 hours 3. Facial skin integrity intact | Only patients who intubated longer than 24 hours, who were not in prone position with ETTs fixation were included | No obvious limitation noticed | Intubated within 12 hours of admission  **Exclusion:**  Intubated greater than 12 hours prior to admission to MICU, had oral mucosa or facial skin break down prior to enrolment, required nasotracheal intubation, had a documented allergy to tape, were pregnant, were prisoners | Over 18 years, no existing oral or nasal mucosal and skin injuries.  ETT or NGT inserted no more than 24 hours prior to enrolment, expected to remain in ICU for more than 48 hours.  **Excluded:**  Had a ETT wired in place, extensive burns, facial trauma or traumatic brain injury requiring intracranial pressure monitoring and /or ventricular drainage of CSF. Cervical spine injured patients with a hard collar in situ, hirsute patients with beard to apply fixation was not possible. Treatment was withdrawn or were being palliated | First mechanical ventilation during current hospitalization, no existing facial wounds or pressure ulcers at time of admission to ICU and intubation performed in the ICU or within two hours prior to ICU arrival. | Patients aged 18-65 years were on orotracheal intubated, had no face and neck trauma, had no burns on the face, had no oral pressure ulcers, were connected to a mechanical ventilator, had no DM and gave verbal and written consent. |
| **Methods of endotracheal security:** | **Lillihei harness:**  Strips of waterproof tape are applied around the patients face above and below the lips and around the ETTs to keep it in place  **Comfit holder**:  A cloth band is placed under the patient’s neck. A double loop of vinyl tubing is passed over the adapter of the ETTs and pulled tightly; in addition, Velcro tabs are attached to the outside of the neckband to hold the ETTs securely.  **Dale holder:**  Adhesive strip is applied around the ETTs. A cloth band is wrapped around the adhesive and then around the patient’s face, crisscrossing both ends in back of the head and pressing the fastener tabs to the band.  **Secure Easy ETT holder:**  By means of bite block and tube holder, a padded faceplate and two nonelastic head straps. | **Fixsond method versus AnchorFast:**  Detailed not mentioned | **AnchorFast:**  Gliding tube: enables the ETT to be easily repositioned while being held securely in place  Lip stabiliser:  Prevents the ETT from resting on the patient’s upper lip.  Contraindicated in patients without teeth, with facial edema, or with protruding teeth, facial hair, profuse diaphoresis, or allergic reaction to the device’s skin barrier pads. | **Adhesive tap versus AnchorFast:**  Detailed not mentioned | **AnchorFast:** ETT attachment device. Mepilex Lite was used to pad the mouth where the ETT was located.  Cotton tape:  Secured around the ETT and tied around participants’ neck using foam to protect the skin at the nape of the neck and the cheeks. Barrier wipe was applied to the skin around the mouth, and Mepilex Lite was used to pad the mouth where the ETT was located. | **Cloth tap versus AnchorFast:**  Detailed not mentioned | **Holder versus**  **Bandage**  Detailed not mentioned |
| **Etiology to ICUs** | Medical or surgical complications related to cancer treatment | No information | No information | Indication for intubation:  Respiratory failure (H/TSM: 70/77), altered mental status (36/31), airway patency (45/35), shock (2/2). | Include trauma (general or neurological), burns, acute respiratory distress, sepsis, multiple organ failure, and complex surgical cases, overdoses, and short term post-operative cases. | Pulmonary or respiratory emergency, septic shock, emergency neurological/neurosurgical conditions(stroke/neurosurgery), Trauma, post-surgical recovery, Emergency cardiac condition (MI), Haematological condition, post-CPR. | **Internal medicine, chest disease, general surgery, cardiology** |
| **Care protocol** | Evaluation signs of injury of tongue and inside of the mouth or lips every 12 hours except Lillihei harness group | ICU nurses checked lips daily to ascertain whether there were signs of pressure ulcers. | Cloth tape changed and ETT repositioned every 6 hours, or when the cloth is soiled; ETTs should be repositioned every 2 hours for patients with AnchorFast in situ.  AnchorFast devices should be replaced every 3-5 days | Repositioned was done as needed according to ICU policy or protocols or at the discretion of the provider; bedside nurse, or respiratory therapist, per usual care.  Oral hygiene was performed every 12 hours and oral moistening every 2 hour based on ICU policy. | Device was in place and for a further 72 hours after evidence removal if the patient remained in the ICU.  ; ETT: 12 h-hourly reposition.  Mean number of ETT reposition in a 48-hour period was 14.4. times/48-hours in holder group, 4.7 times/48-hours in non-holder group. | **Cloth tape:**  Daily tape replacement, gauze pads on pressure sites (ears, lips, cheeks) were used as a method of routine protection from PU.  **AnchorFast:**  Does not required daily change, according to researcher observation | Followed up for 4 days in terms of oral pressure ulcers from the day intubated. Tube fixation of both groups was renewed every 24 hours and the tube was repositioned every four hours.  At the end of the 4^th^ day, wound was evaluation according to international staging system for pressure injuries and the pressure ulcer scale for healing. |
| **Number of days patients in the study** | (1-23 days), Median  3 ± 3.9 days | No information, ETTs intubated day had no different between two groups | *****Length of mechanical ventilation days:  Tape: 7.1 (3.8-16.7)  AnchorFast: 7.9 (3.6-13.9)  **Time from intubation to PI reporting days:**  Tape: 3 (1-5.5), AnchorFast: 4 (2-9) | Length of mechanical ventilation days:  Holder: 3.9 ± 3.0  TSM: 3.9 ± 3.4 | Median:  8.38 in ICU  (5.41-11.69) days | Hospital days until PU formation: H/NH: 15.29/6.07  Ventilation days until PU formation: 13.86/5.04 | Mean duration for being intubated in the ICU: 1.15 ± 0.44, minimum: 1 day,  Maximum: 3 days |
| **Pressure ulcer evaluation** | Signs of injury to tongue, inside the mouth or on lips, rating facial skin integrity made on a scale of 0 to 3.  **The scale detailed not provided.** | Only detect grade II or high-grade pressure ulcers | The date and time of ulcer documentation, severity and location were retrieved (corner of mouth, lip or mouth) | Not divided to severity, include lip ulcer, skin tear | Number Location, size, staging and attributable device were recorded., staging according to international PI classification system of four stages.  **The staging detailed not provided** | Site of PU development, PU grade, time from intubation and hospitalisation in ICU.  Include ear, lip and cheek area. | At the end of the 4^th^ day, wound was evaluation according to international staging system for pressure injuries and the pressure ulcer scale for healing.  **The staging detailed not provided** |
| **BMI** | NA | NA | NA | H: 30.3 ± 10.7  TSM: 30.8 ± 11.1 | Median*: 29 (24–37) | NA | 26.85 ± 2.46 (total) |
| **Albumin** | NA | NA | Albumin in pressure injury patients: H/ TSM: 32 (27.5–36.0)/36 (27.0–39.5) | NA | NA | Albumin on admission (g/L): H: 29.87 ± 7.12  TSM: 29.18 ± 6.84 | NA |
| **DM** | NA | NA | H/ TSM: 1(4.8%)/5(23.8%) | NA | NA | H/ TSM: 1(4.8%)/5(23.8%) | NA |
| **Peripheral Artery Disease** | NA | NA | H/ TSM: 1(4.8%)/0(0%) | NA | 4 (8.9%)* | NA | Total 19 patients had cardiovascular disease |
| **Current Smoker** | NA | NA | H/ TSM: 7(33.3%)/4(19%) | NA | 14 (31.8%)* | NA | H/NH: 16/17 |
| **Steroid Use** | NA | NA | H/ TSM: 5(23.8%)/2(9.5%) | NA | NA | NA | H/NH: 21/21 |
| **Vasopressor Use** | NA | NA | H/ TSM: 8/7 | NA | NA | NA | H/NH: 29/26 |
| **Others** |  | Only detect grade II or higher-grade pressure ulcers | ***Restricted mobility:**  **H/TSM: 5/6** | Defined dislodgement as needing to reposition the ETTs more than 1 cm. | Lower reposition ETT rate (47%) and low rates of application of Mepilex Lite to the skin surface surrounding the device (33%) |  | **Provide the correlation of coma status and ulcer occurrence** |

*Only patients with pressure injuries (PI):

NA, Not Available, H, Holder, TSM, Traditional securing method.

**Table S5. Detailed outcome of the included studies**

| **Study** | **Kaplow 1994** | **Embregts 2011** | **Hampson 2018** | **Landsperger 2019** | **Coyer 2020** | **Kuniavsky 2020** | **Genc 2021** |
| --- | --- | --- | --- | --- | --- | --- | --- |
| **Pressure injury events (Holder/Traditional securing methods)** | | | | | | | |
| **Lip ulcers** | **14/11** | **3/12** | **23/24** | **4/11** | **No information** | **4/6** | **No information** |
| **Oral ulcers** | **No information** | **No information** | **1/4** | **No information** | **No information** | **No information** | **29/26** |
| **Face tears** | **4/4** | **No information** | **No information** | **2/3** | **No information** | **2/0** | **No information** |
| **Others** | **Back of neck:**  **5/2** | **No information** | **No information** | **No information** | **No information** | **Right ear: 0/9**  **Both ear: 0/9**  **Right ear and lip: 0/2**  **Left ear: 1/18**  **Left ear and lip: 0/1** | **No information** |
| **Total number** | **23/17** | **3/12** | **24/28** | **6/14** | **0/1** | **7/45** | **29/26** |
| **Tube dislodge composite events (Holder/ Traditional securing methods)** | | | | | | | |
| **Tube dislodge > 1 cm** | **Comfit: 9**  **Dale: 6**  **Secure easy: 3**  **Lillihei: 9** | **No information** | **No information** | **6/15** | **No information** | **No information** | **No information** |
| **Self-extubation** | **No mentioned** | **9/9** | **No information** | **2/2** | **No information** | **No information** | **No information** |
| **Total** | **18/9** | **9/9** | **No information** | **8/17** | **No information** | **No information** | **No information** |
| **Total patient**  **(Holder/ Traditional securing methods)** | **81/30** | **80/69** | **596/1412** | **250/250** | **21/21** | **77/78** | **30/30** |

**(A)**
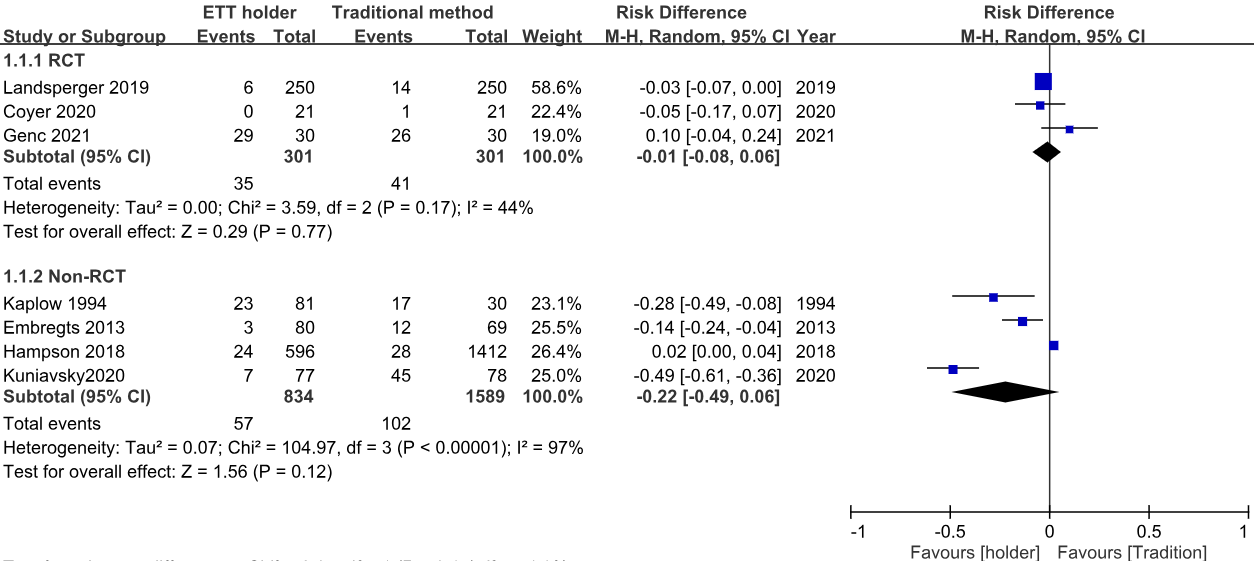


**(B)**
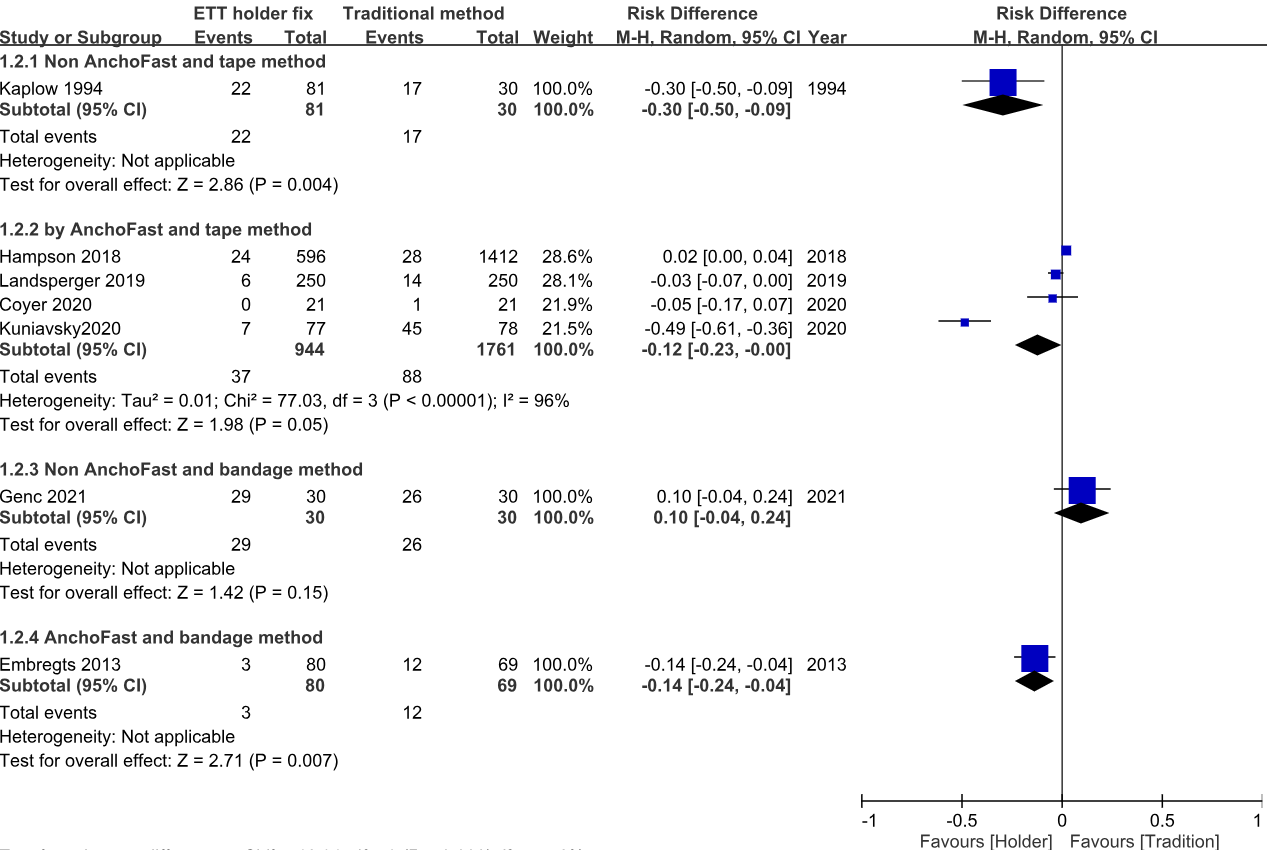


**(C)**
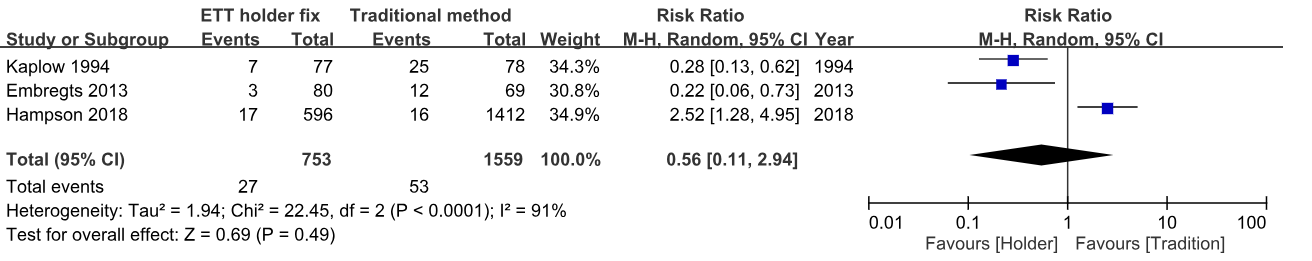


(D)
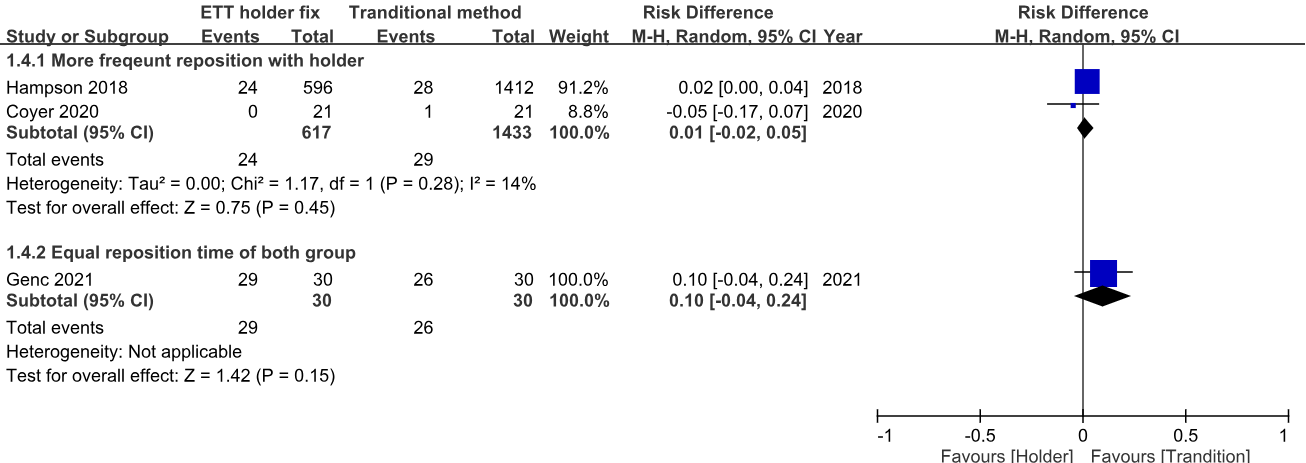


**Figure S1. Subgroup analysis of total medical device-related pressure injury (MDRPI) events by: (A) Study type (B) Type of holder vs traditional securing methods (C) Pressure injuries above grade 2 only (D) Frequency of repositioning**


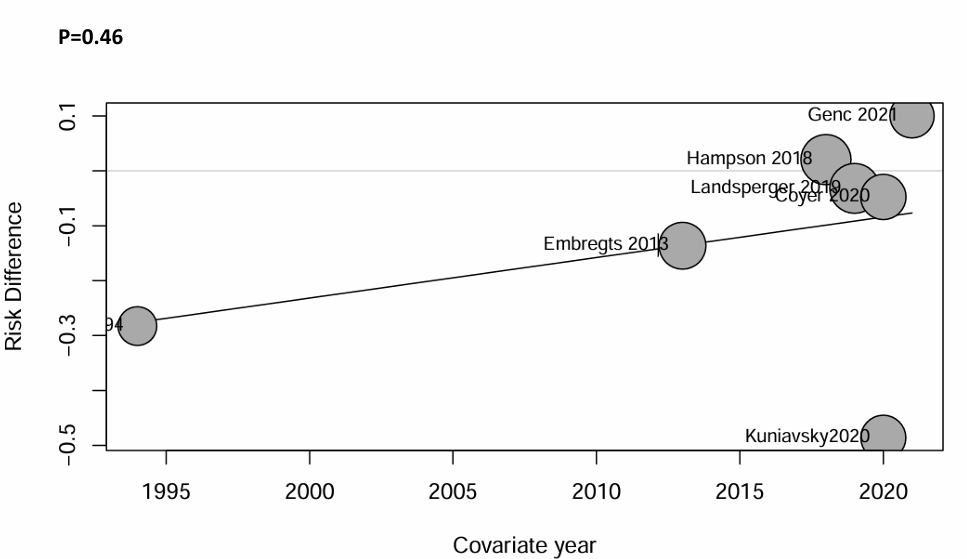


**(A)**

**
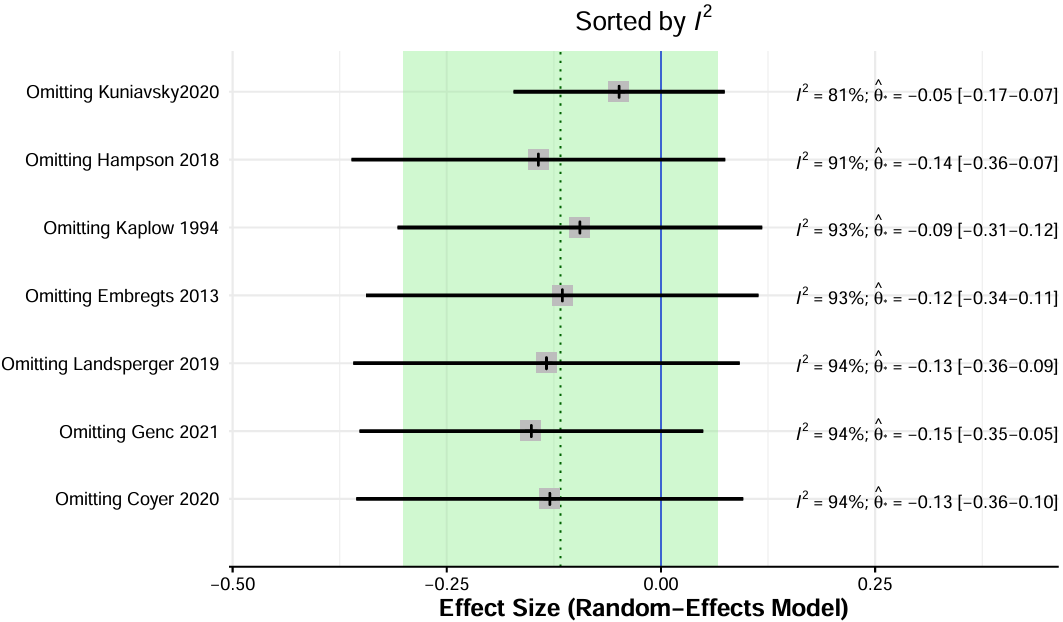
**

**(B)**

**
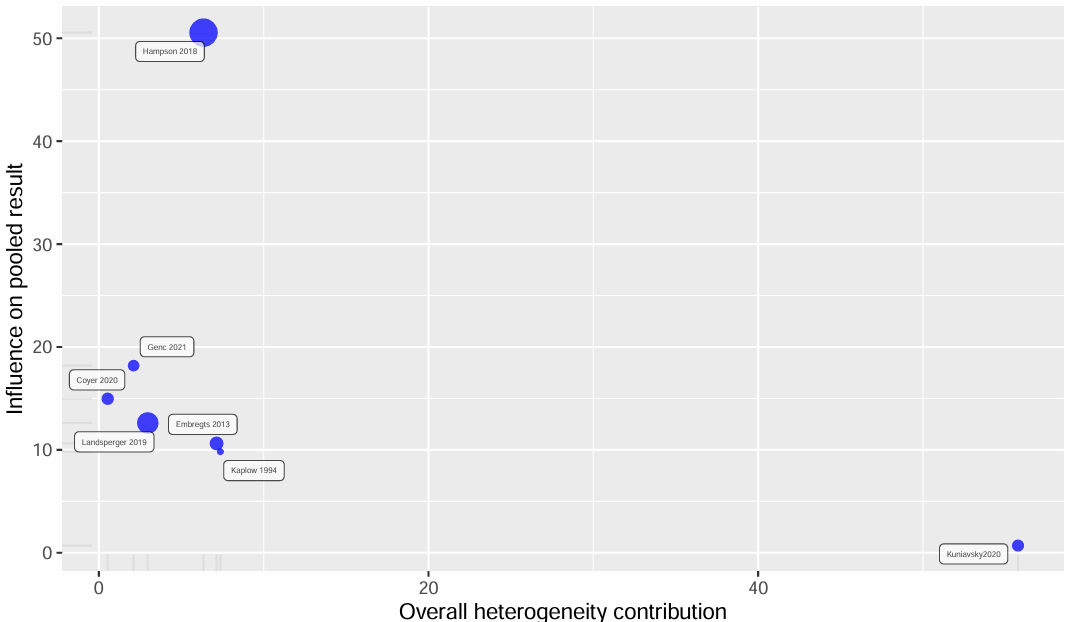
**

**(C)**

**Figure S2. (A) Meta-regression by publication year (B) Sensitivity analysis (C) Influence analysis of total medical device-related pressure injuries (MDRPIs)**

**
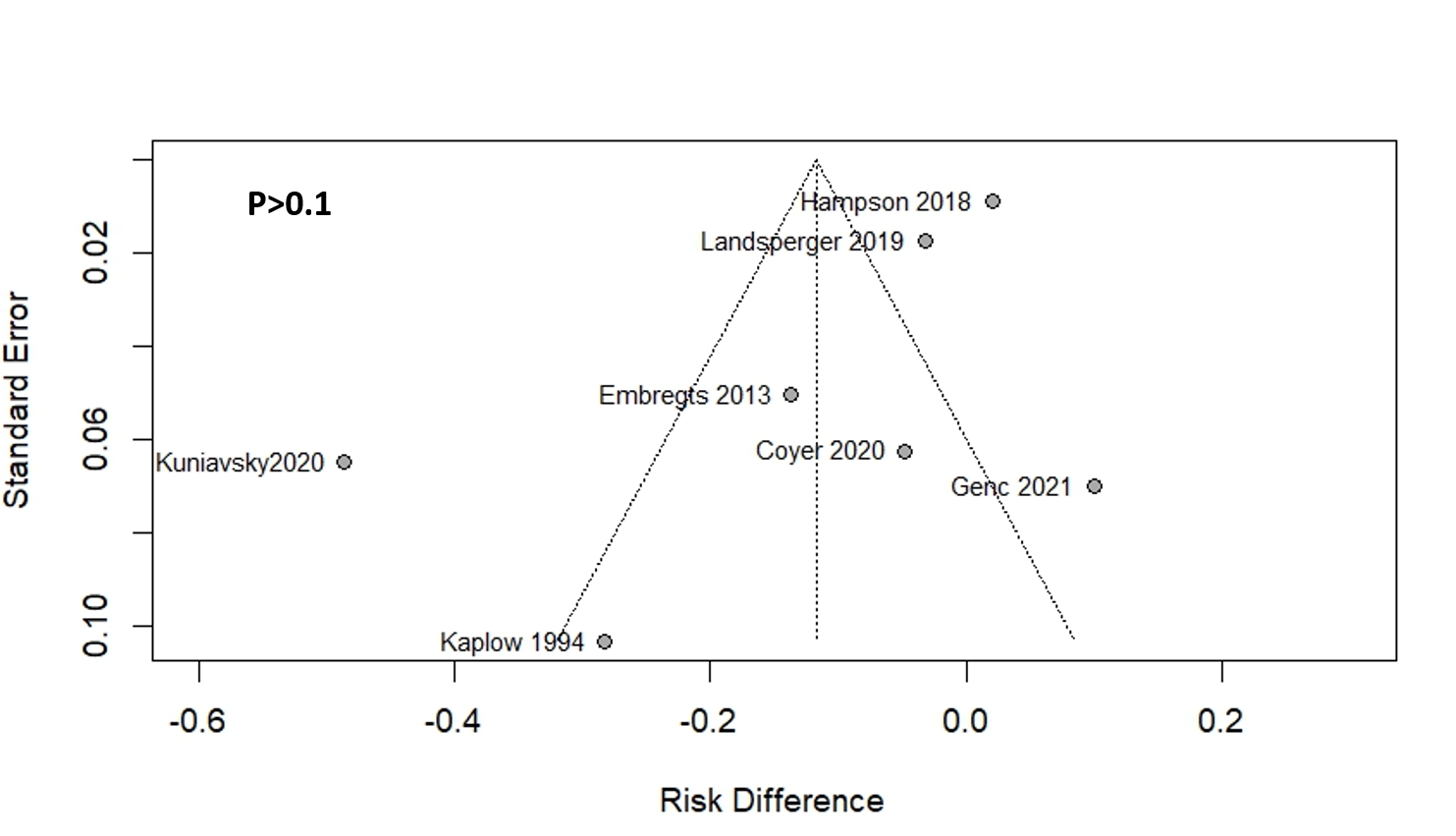
**

**Figure S3. Funnel plot of included studies (N = 7)**

**
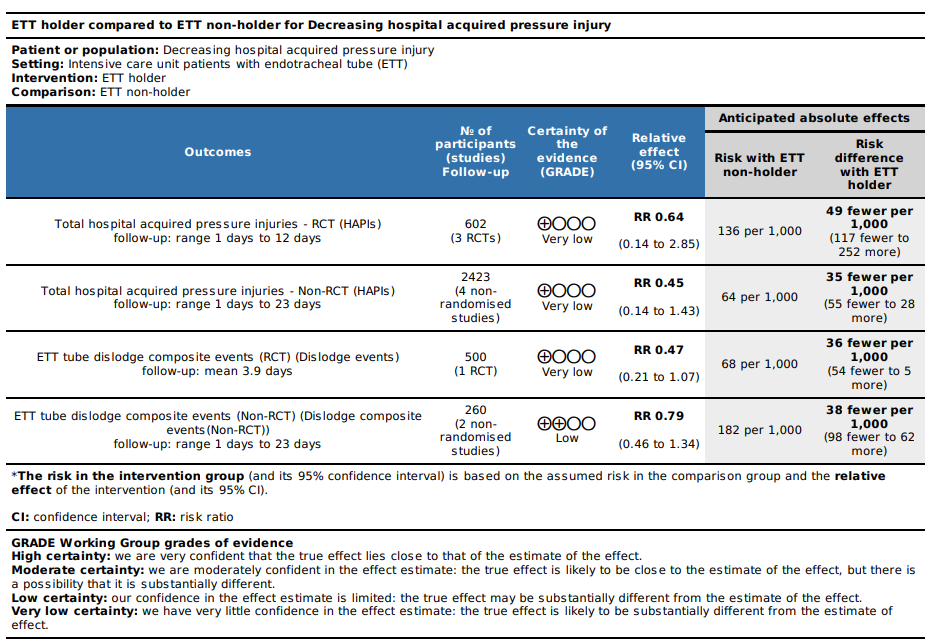
**

**Figure S4. GRADE evidence table for patients in Intensive Care Units receiving endotracheal tube (ETT) holders versus traditional securing methods (non-holders)**

**GRADE, Grading of Recommendations, Assessment, Development, and Evaluation**
